# Sex Disparities in Management and Outcomes Among Patients With Acute Coronary Syndrome

**DOI:** 10.1001/jamanetworkopen.2023.38707

**Published:** 2023-10-20

**Authors:** Shuduo Zhou, Yan Zhang, Xuejie Dong, Xu Zhang, Junxiong Ma, Na Li, Hong Shi, Zuomin Yin, Yuzeng Xue, Yali Hu, Yi He, Bin Wang, Xiang Tian, Sidney C. Smith, Ming Xu, Yinzi Jin, Yong Huo, Zhi-Jie Zheng

**Affiliations:** 1 Department of Global Health, Peking University School of Public Health, Beijing, China; 2 Institute for Global Health and Development, Peking University, Beijing, China; 3 Division of Cardiology, Peking University First Hospital, Beijing, China; 4 Peking Union Medical College Hospital, Chinese Academy of Medical Science and Peking Union Medical College, Beijing, China; 5 Chinese Medical Association, Beijing, China; 6 Department of Emergency, The Affiliated Qingdao Central Hospital of Qingdao University, The Second Affiliated Hospital of Medical College of Qingdao University, Qingdao, Shandong, China; 7 Division of Cardiology, Liaocheng People’s Hospital, Liaocheng, China; 8 Division of Cardiology, Cangzhou People’s Hospital, Cangzhou, China; 9 Division of Cardiology, Zhuzhou Central Hospital, Zhuzhou, China; 10 Division of Cardiology, First Affiliated Hospital of Shantou University Medical College, Shantou, China; 11 Division of Cardiology, Baoding No.1 Central Hospital, Baoding, China; 12 Division of Cardiovascular Medicine, School of Medicine, The University of North Carolina at Chapel Hill

## Abstract

**Importance:**

Sex disparities in the management and outcomes of acute coronary syndrome (ACS) have received increasing attention.

**Objective:**

To evaluate the association of a quality improvement program with sex disparities among patients with ACS.

**Design, Setting, and Participants:**

The National Chest Pain Centers Program (NCPCP) is an ongoing nationwide program for the improvement of quality of care in patients with ACS in China, with CPC accreditation as a core intervention. In this longitudinal analysis of annual (January 1, 2016, to December 31, 2020) cross-sectional data of 1 095 899 patients with ACS, the association of the NCPCP with sex-related disparities in the care of these patients was evaluated using generalized linear mixed models and interaction analysis. The robustness of the results was assessed by sensitivity analyses with inverse probability of treatment weighting. Data were analyzed from September 1, 2021, to June 30, 2022.

**Exposure:**

Hospital participation in the NCPCP.

**Main Outcomes and Measures:**

Differences in treatment and outcomes between men and women with ACS. Prehospital indicators included time from onset to first medical contact (onset-FMC), time from onset to calling an emergency medical service (onset-EMS), and length of hospital stay without receiving a percutaneous coronary intervention (non-PCI). In-hospital quality indicators included non-PCI, use of statin at arrival, discharge with statin, discharge with dual antiplatelet therapy, direct PCI for ST-segment elevation myocardial infarction (STEMI), PCI for higher-risk non–ST-segment elevation ACS, time from door to catheterization activation, and time from door to balloon. Patient outcome indicators included in-hospital mortality and in-hospital new-onset heart failure.

**Results:**

Data for 1 095 899 patients with ACS (346 638 women [31.6%] and 749 261 men [68.4%]; mean [SD] age, 63.9 [12.4] years) from 989 hospitals were collected. Women had longer times for onset-FMC and onset-EMS; lower rates of PCI, statin use at arrival, and discharge with medication; longer in-hospital delays; and higher rates of in-hospital heart failure and mortality. The NCPCP was associated with less onset-FMC time, more direct PCI rate for STEMI, lower rate of in-hospital heart failure, more drug use, and fewer in-hospital delays for both men and women with ACS. Sex-related differences in the onset-FMC time (β = −0.03 [95% CI, −0.04 to −0.01), rate of direct PCI for STEMI (odds ratio, 1.11 [95% CI, 1.06-1.17]), time from hospital door to balloon (β = −1.38 [95% CI, −2.74 to −0.001]), and rate of in-hospital heart failure (odds ratio, 0.90 [95% CI, 0.86-0.94]) were significantly less after accreditation.

**Conclusions and Relevance:**

In this longitudinal cross-sectional study of patients with ACS from hospitals participating in the NCPCP in China, sex-related disparities in management and outcomes were smaller in some aspects by regionalization between prehospital emergency and in-hospital treatment systems and standardized treatment procedures. The NCPCP should emphasize sex disparities to cardiologists; highlight compliance with clinical guidelines, particularly for female patients; and include the reduction of sex disparities as a performance appraisal indicator.

## Key Points

**Question:**

Is there an association between health care quality improvement programs and sex disparities among patients with acute coronary syndrome (ACS)?

**Findings:**

In this cross-sectional study, 1 095 899 patients with ACS were analyzed. The findings highlight significant sex-related differences in the quality of services for ACS by regionalization of prehospital emergency and in-hospital treatment systems, standardization of treatment procedures, and health education at the community level.

**Meaning:**

These findings suggest that the concept of sex differences should be included in health care quality improvement programs from a systemic perspective to further reduce the sex disparities in patients with ACS.

## Teaser

This cross-sectional study assesses sex-related differences in the quality of health care services for patients with acute coronary syndrome (ACS) in China and whether the National Chest Pain Centers Program (NCPCP) is associated with improved disparities.

## Introduction

Health equity has been at the core of the sustainable development agenda, with both the United Nations millennium development goals and sustainable development goals now including promotion of equity and reduction of disparities as critical development goals.^1,2^ Health inequity refers specifically to differences or disparities in quality of health care between different groups of people.^3^ Quality of health care includes quality of services used and treatment processes and outcomes.^4^ Inequalities in quality of service stemming from differences in use of health services are a crucial factor contributing to inequalities in health outcomes.^5^

There are significant sex-related disparities in the use of health care services and clinical outcomes in patients with acute coronary syndromes (ACS).^6,7,8^ Women with ACS are significantly more likely to have delayed access to care and are less likely to receive aggressive invasive therapy and medication, thereby having poorer clinical outcomes than their male counterparts.^9,10,11,12^ The proportions of women with ACS who receive early dual antiplatelet therapy (DAPT) (89.0% vs 93.5%) and reperfusion therapy (50.2% vs 59.5%) and those who are discharged with DAPT (82.8% vs 90.1%) are reportedly lower than those of men, and in-hospital mortality rate is significantly higher in women (2.6% vs 1.5%).^13^ Furthermore, women with ACS often present with atypical symptoms, such as vomiting. Therefore, women are less likely to undergo diagnostic tests, such as electrocardiography or troponin level measurement, and clinical guidelines for women with ACS are inadequate in terms of recognizing cardiovascular disease.^14,15,16,17^

Reducing inequities in quality of service provision can reduce morbidity and disparities in service use in patients with ACS.^18^ Quality improvement programs may improve inequities in health care,^19,20^ including the areas of maternal and child health,^21^ cancer,^22^ and cardiovascular disease.^23^ Quality improvement programs had improved treatment-related disparities between White patients with acute myocardial infarction and patients of other races and ethnicities in the US.^24,25^ However, whether these programs can identify women with ACS in a timely manner and reduce sex-related disparities in their care needs further investigation.^26^ The Chinese Cardiovascular Association launched the National Chest Pain Centers Program (NCPCP) in January 2016. The aims of this nationwide, hospital-based quality improvement program are to establish regional emergency care networks and improve early diagnosis and treatment of patients with ACS.^27^ The NCPCP includes the following 5 components: accreditation of hospital-based CPCs, performance measure and assessment, quality audit and feedback, training for clinicians, and education for the community (eTable 1 in Supplement 1). As of May 2021, 1927 hospitals in 31 provinces in China had a certified CPC and over 5000 hospitals have joined the NCPCP (eFigure 1 in Supplement 1).

Although sex-related disparities in health care have been an increasing focus of attention, few studies have investigated how these disparities can be reduced.^20,28^ The main objectives of this study were to determine whether there were sex-related differences in the quality of health care services for patients with ACS in China and, if so, whether the NCPCP had improved these disparities.

## Methods

Ethical approvals from the NCPCP were obtained from the institutional review boards of the Gusu group ethics committee. Informed consent was obtained from the registered hospitals for research approval to collect data in the program. Our study protocol was approved by the Ethics Committee of Peking University Health Science Center. The need for informed consent was waived by the Ethics Committee of Peking University Health Science Center owing to the use of deidentified data. This study followed the Strengthening the Reporting of Observational Studies in Epidemiology (STROBE) reporting guideline.

### Study Design

We used a longitudinal self-contrast cross-sectional comparison design that included hospitals that had acquired NCPCP accreditation and had all 3 accreditation statuses: before, undergoing, and after accreditation. All the data in this study were from the hospitals that had successfully accredited with NCPCP to ensure consistency in the composition of hospitals before, during, and after accreditation. The design and implementation of the NCPCP have been described in detail elsewhere.^27^ The NCPCP requires registered hospitals to enroll consecutive cases presenting with ACS. All the registered hospitals are responsible for collecting their own data and reporting the data of patients with ACS to the Chinese Cardiovascular Association (CCA) Database–CPC. Three levels of experts, including national, provincial, and prefecture, reviewed the reported data for completeness and accuracy.

All hospitals must upload their data about patients with ACS for at least 6 months before formal accreditation. For on-site accreditation evaluation, the CPC Accreditation Board reviews the data within 6 months and the achievement of each index in the last 6 months. Therefore, the time between the on-site assessment and the previous 6 months can be considered as the time during accreditation. Patients who were admitted to a hospital 6 months or more before the accreditation date were coded as the before accreditation group; those who were admitted to a hospital during the 6 months before the accreditation date, the undergoing accreditation group; and those who were admitted to a hospital after the date of accreditation, the after accreditation group.^29^

### Data Collection

The data for this study were obtained from the CCA Database–CPC. Data from hospitals accredited before January 1, 2016, patients discharged prior to 2016, those who did not meet requirements for statistical analysis (eg, missing sex), and those from hospitals that had fewer than 50 cases before, during, and after accreditation were excluded.^30^ We analyzed the final data for 1 095 899 inpatients with ACS from January 1, 2016, to December 31, 2020. Among these patients, 457 921 had ST-segment elevation myocardial infarction (STEMI) and 637 978 had non–ST-segment elevation ACS (NSTE-ACS) (eFigure 2 in Supplement 1). Information of participant sex was obtained using self-report survey measures, and information on ethnicity was not included in the survey.

### Study Variables

#### Outcome Indicators

The quality evaluation dimension was guided by the input process–output framework and quality of care as defined by Campbell et al.^31^ The variables used to determine whether there were any differences in outcomes between men and women with ACS were selected based on the clinical guidelines for STEMI and NSTE-ACS and the indicators used by the NCPCP to evaluate quality of care. Prehospital quality indicators included time from onset to first medical contact (onset-FMC), time from onset to calling an emergency medical service (onset-EMS), and length of hospital stay without receiving percutaneous coronary intervention (non-PCI). In-hospital quality indicators included non-PCI, use of statin at arrival, discharge with statin, discharge with DAPT, direct PCI for STEMI, PCI for higher-risk NSTE-ACS, time from door to catheterization activation, and time from door to balloon. Patient outcome indicators included in-hospital mortality and in-hospital new-onset heart failure (eTable 2 in Supplement 1).

#### Covariates

Information about patient demographic variables, vital baseline characteristics at onset, severity of illness, and hospital-level characteristics was used to control for potential confounding. These variables included age, sex, consciousness level (conscious, responsive to speech, responsive to tingling, unresponsive to stimulus, or missing), onset respiration (12-24 breaths/min, other, or missing), onset pulse (60-100 beats/min [bpm], other, or missing), onset heart rate (60-100 bpm, other, or missing), onset hypertension (yes, no, or missing), chest tightness or chest pain (yes or no), Killip classification (I-IV), hospital level (secondary or tertiary), type of accreditation (basic or standard), patient arrival mode (walk-in, transported by EMS, interhospital transfer, or onset in hospital), and region (eastern, middle, or western China).

### Statistical Analysis

Descriptive statistics were used to describe the demographic characteristics of the study sample. The Pearson χ^2^ and Wilcoxon rank sum tests were used to compare categorical and continuous variables, which are summarized as the percentage and median (IQR), respectively. We used multilevel mixed-effects regression models to examine the association of NCPCP with the quality of service provision for male and female patients with ACS, with adjustment for clustering at the hospital level and allowing for hospital-level estimates as random effects.^32,33^ The statistical models were evaluated for goodness of fit using likelihood ratio tests. Logistic regression models were used for binary outcomes and generalized linear models with a log or identity link for continuous outcomes. Time-fixed effects were added to the models to control for time trends and the effects of other policies. Interactions between accreditation status (before, undergoing, or after) and sex (male or female) were added to the models to analyze the association of NCPCP with sex differences in quality of service. The coefficient and *P* value for sex and accreditation status interaction terms in these models indicate the direction of change in sex-related disparities in quality of health care and whether the change is significant. In view of the common problem of heteroskedasticity in cross-sectional data, the heteroskedasticity-robust standard error was used to ensure the stability of coefficients.^34^

To maximize statistical efficacy, the sample size in our regression model varied with the number of dependent variables^35^ (eTable 3 in Supplement 1). For some of the missing control variables (<6%; for the Killip classification, about 15%), the missing data due to reasons such as omission in data collection were coded as a separate category for each covariate.^36^ Sensitivity analysis was used to test the robustness of the generalized linear mixed-model estimators. Inverse probability of treatment weighting (IPTW) was used to eliminate differences in baseline characteristics of individuals in the before, undergoing, and after accreditation groups. In the IPTW method, weights are assigned to patients based on the inverse of their probability of receiving the NCPCP intervention. The statistical analyses were performed using Stata, version 14.1 (StataCorp LLC), and R Studio, version 1.2.5042 (R Project for Statistical Computing). All tests were 2-sided and *P* < .05 was considered statistically significant.

## Results

The study included 1 095 899 patients with ACS (749 261 men [68.4%] and 346 638 women [31.6%]; mean [SD] age, 63.9 [12.4] years) from 989 hospitals. Of these patients, 256 682 were in the before accreditation group (175 002 men [68.2%] and 81 680 women [31.8%]), 216 365 were in the undergoing accreditation group (144 763 men [66.9%] and 71 602 women [33.1%]), and 622 852 were in the after accreditation group (429 496 men [69.0%] and 193 356 women [31.0%]). Almost all patients were conscious and breathing at the time of onset. Women were less likely to have hypertension at the time of onset, more likely to be in Killip class III or IV, and less likely to arrive at hospital within 120 minutes or by interhospital transfer. The proportion of men with ACS in tertiary care hospitals (11.3%) was significantly higher than that of women (10.9%) (*P* < .001) (Table 1).

**Table 1.** Characteristics of Participants With ACS Stratified by Hospital Accreditation Status

Characteristic	Accreditation classification, No. (%)^a^					
	Before		Undergoing		After	
	Men (n = 175 002)	Women (n = 81 680)	Men (n = 144 763)	Women (n = 71 602)	Men (n = 429 496)	Women (n = 193 356)
Age, y						
18-39	7441 (4.3)	571 (0.7)	6485 (4.5)	575 (0.8)	20 051 (4.7)	1343 (0.7)
40-59	70 550 (40.3)	17 090 (20.9)	58 770 (40.6)	15 354 (21.4)	177 179 (41.3)	40 008 (20.7)
60-79	84 516 (48.3)	52 127 (63.8)	69 277 (47.9)	45 071 (62.9)	203 787 (47.4)	124 119 (64.2)
≥80	12 495 (7.1)	11 892 (14.6)	10 231 (7.1)	10 602 (14.8)	28 479 (6.6)	27 886 (14.4)
Onset consciousness						
Conscious	168 472 (96.3)	78 902 (96.6)	141 318 (97.6)	70 006 (97.8)	420 162 (97.8)	189 470 (98.0)
Responsive to speech	526 (0.3)	330 (0.4)	318 (0.2)	249 (0.3)	884 (0.2)	557 (0.3)
Responsive to tingling	363 (0.2)	259 (0.3)	295 (0.2)	165 (0.2)	664 (0.2)	392 (0.2)
Unresponsive to stimulus	352 (0.2)	138 (0.2)	283 (0.2)	132 (0.2)	637 (0.1)	247 (0.1)
Missing	5289 (3.0)	2051 (2.5)	2549 (1.8)	1050 (1.5)	7149 (1.7)	2690 (1.4)
Onset respiration rate						
12-24 breaths/min	166 679 (95.2)	77 555 (94.9)	139 590 (96.4)	68 664 (95.9)	412 839 (96.1)	185 183 (95.8)
Others	4550 (2.6)	2689 (3.3)	3532 (2.4)	2288 (3.2)	9414 (2.2)	5544 (2.9)
Missing	3773 (2.2)	1436 (1.8)	1641 (1.1)	650 (0.9)	7243 (1.7)	2629 (1.4)
Onset pulse						
60-100 bpm	134 656 (76.9)	63 233 (77.4)	111 185 (76.8)	55 142 (77.0)	327 593 (76.3)	148 718 (76.9)
Others	36 361 (20.8)	16 863 (20.6)	31 414 (21.7)	15 539 (21.7)	91 361 (21.3)	40 587 (21.0)
Missing	3985 (2.3)	1584 (1.9)	2164 (1.5)	921 (1.3)	10 542 (2.5)	4051 (2.1)
Onset heart rate						
60-100 bpm	134 461 (76.8)	62 891 (77.0)	111 099 (76.7)	54 952 (76.7)	330 897 (77.0)	149 761 (77.5)
Others	37 229 (21.3)	17 462 (21.4)	32 102 (22.2)	16 058 (22.4)	93 898 (21.9)	42 046 (21.7)
Missing	3312 (1.9)	1327 (1.6)	1562 (1.1)	592 (0.8)	4701 (1.1)	1549 (0.8)
Onset hypertension						
Yes	89 568 (51.2)	37 065 (45.4)	72 653 (50.2)	31 558 (44.1)	216 732 (50.5)	87 410 (45.2)
No	82 682 (47.2)	43 556 (53.3)	70 757 (48.9)	39 502 (55.2)	208 783 (48.6)	104 628 (54.1)
Missing	2752 (1.6)	1059 (1.3)	1353 (0.9)	542 (0.8)	3981 (0.9)	1318 (0.7)
Killip class						
I	121 934 (69.7)	52 343 (64.1)	105 292 (72.7)	48 340 (67.5)	311 377 (72.5)	129 992 (67.2)
II	18 528 (10.6)	9461 (11.6)	12 741 (8.8)	7258 (10.1)	48 592 (11.3)	25 300 (13.1)
III	6161 (3.5)	3848 (4.7)	3606 (2.5)	2711 (3.8)	13 647 (3.2)	9271 (4.8)
IV	5296 (3.0)	2769 (3.4)	3904 (2.7)	2148 (3.0)	13 235 (3.1)	6920 (3.6)
Missing	23 083 (13.2)	13 259 (16.2)	19 220 (13.3)	11 145 (15.6)	42 645 (9.9)	21 873 992 (11.3)
Chest pain and/or tightness						
Persistent	86 340 (49.3)	30 936 (37.9)	67 587 (46.7)	26 268 (36.7)	211 732 (49.3)	74 459 (38.5)
Intermittent	67 611 (38.6)	39 485 (48.3)	58 194 (40.2)	34 809 (48.6)	169 107 (39.4)	94 725 (49.0)
Symptom relief	12 486 (7.1)	5848 (7.2)	10 768 (7.4)	5150 (7.2)	29 334 (6.8)	12 499 (6.5)
Missing	8565 (4.9)	5411 (6.6)	8214 (5.7)	5375 (7.5)	19 323 (4.5)	11 673 (6.0)
Patient arrival mode						
EMS transport	17 107 (9.8)	7774 (9.5)	13 445 (9.3)	6925 (9.7)	35 251 (8.2)	15 679 (8.1)
Interhospital transfer	29 343 (16.8)	9634 (11.8)	24 770 (17.1)	8934 (12.5)	82 885 (19.3)	27 559 (14.3)
Walk-in	125 589 (71.8)	62 600 (76.6)	103 579 (71.6)	54 206 (75.7)	304 311 (70.9)	146 545 (75.8)
In-hospital onset	2954 (1.7)	1666 (2.0)	2964 (2.0)	1535 (2.1)	7021 (1.6)	3555 (1.8)
Missing	9 (0.01)	6 (0.01)	5 (0.003)	2 (0.003)	28 (0.01)	18 (0.01)
Hospital level						
Secondary	50 275 (28.7)	27 908 (34.2)	40 786 (28.2)	23 676 (33.1)	102 556 (23.9)	56 063 (29.0)
Tertiary	20 606 (11.8)	9847 (12.1)	16 394 (11.3)	7785 (10.9)	44 199 (10.3)	19 450 (10.1)
Grade IIIA	104 121 (59.5)	43 925 (53.8)	87 583 (60.5)	40 141 (56.1)	282 741 (65.8)	117 843 (60.9)
Accreditation type^b^						
Basic	41 406 (23.7)	23 049 (28.2)	33 326 (23.0)	19 767 (27.6)	79 099 (18.4)	43 708 (22.6)
Standard	133 596 (76.3)	58 631 (71.8)	111 437 (77.0)	51 835 (72.4)	350 397 (81.6)	149 648 (77.4)
Admission year						
2016	11 838 (6.8)	4833 (5.9)	1666 (1.2)	797 (1.1)	23 (0.01)	6 (0.003)
2017	46 382 (26.5)	21 320 (26.1)	29 404 (20.3)	13 635 (19.0)	13 702 (3.2)	6067 (3.1)
2018	67 376 (38.5)	32 160 (39.4)	49 334 (34.1)	25 172 (35.2)	72 522 (16.9)	32 450 (16.8)
2019	40 108 (22.9)	18 952 (23.2)	49 956 (34.5)	24 992 (34.9)	148 972 (34.7)	68 982 (35.7)
2020	9298 (5.3)	4415 (5.4)	14 403 (9.9)	7006 (9.8)	194 277 (45.2)	85 851 (44.4)
Region						
Eastern	76 868 (43.9)	35 249 (43.2)	60 798 (42.0)	29 771 (41.6)	187 690 (43.7)	81 455 (42.1)
Middle	56 397 (32.2)	29 306 (35.9)	49 902 (34.5)	26 967 (37.7)	147 516 (34.3)	73 292 (37.9)
Western	41 737 (23.8)	17 125 (21.0)	34 063 (23.5)	14 864 (20.8)	94 290 (22.0)	38 609 (20.0)

Abbreviations: ACS, acute coronary syndrome; bpm, beats per minute; EMS, emergency medical service.

^a^ Percentages have been rounded and may not total 100.

^b^ The standard version is appropriate for hospitals with an annual percutaneous coronary intervention (PCI) operation volume of 200 or greater, emergency PCI operation volume of 50 or greater, and emergency PCI operations can be performed 24 hours a day. The basic version is primarily aimed toward hospitals that do not meet the requirements of the standard version.

A comparison of the quality of services received is shown by sex in Table 2. The median onset-FMC time was higher in women than in men (302 [IQR, 100-1684] vs 206 [IQR, 74-1124] min; *P* < .001). Rates of statin use at arrival (73.8% vs 71.3%), discharge with statin (87.9% vs 87.0%), discharge with DAPT (88.7% vs 86.9%), PCI treatment for NSTE-ACS (57.2% vs 43.0%), and direct PCI for STEMI (61.1% vs 53.2%) were higher in men than in women (*P* < .001 for all). Median non-PCI hospital length of stay (90 [IQR, 44-218] vs 108 [IQR, 50-284] min), time from door to catheter laboratory activation (44 [IQR, 24-72] vs 46 [IQR, 26-78] min), and time from door to balloon (88 [IQR, 60-130] vs 92 [62-136] min) was shorter in men than in women (*P* < .001 for all). The rates of in-hospital heart failure (12.3% vs 15.3%) and mortality (1.9% vs 2.7%) were lower in men than in women in the before accreditation group, with similar results in the undergoing accreditation and after accreditation groups.

**Table 2.** Comparison of Quality Indicators Between Male and Female Patients With ACS in Different Accreditation Status Hospitals

Quality indicators	Accreditation classification^a^					
	Before		Undergoing		After	
	Men (n = 175 002)	Women (n = 81 680)	Men (n = 144 763)	Women (n = 71 602)	Men (n = 429 496)	Women (n = 193 356)
Onset-FMC, median (IQR), min	206 (74-1124)	304 (100-1684)	194 (70-934)	272 (94-1450)	206 (76-992)	308 (106-1540)
Onset-EMS, median (IQR), min	58 (18-170)	84 (26-296)	52 (18-160)	74 (24-246)	58 (20-176)	86 (30-316)
Non-PCI stay, median (IQR), min	90 (44-218)	108 (50-284)	88 (44-200)	100 (50-274)	86 (42-198)	100 (48-246)
Statin at arrival	112 329 (73.8)	50 111 (71.3)	94 712 (74.1)	44 386 (71.4)	315 115 (80.6)	138 041 (78.9)
DAPT discharge	12 645 (88.7)	6190 (86.9)	19 639 (89.1)	9593 (87.4)	183 183 (92.8)	80 776 (90.3)
Statin discharge	12 532 (87.9)	6193 (87.0)	19 404 (88.1)	9549 (87.0)	180 217 (91.3)	80 179 (89.7)
PCI for STEMI	47 105 (61.1)	12 659 (53.2)	35 797 (62.5)	10 230 (55.3)	129 080 (70.3)	35 655 (65.1)
PCI for high risk of NSTE-ACS	46 492 (57.2)	20 805 (43.0)	41 807 (55.9)	18 994 (42.1)	128 339 (60.4)	56 403 (46.9)
Door to balloon, median (IQR), min	88 (60-130)	92 (62-136)	70 (44-100)	72 (46-104)	70 (46-100)	72 (48-106)
Door to catheterization laboratory activation, median (IQR), min	44 (24-72)	46 (26-78)	34 (16-54)	34 (16-56)	34 (18-54)	36 (18-56)
In-hospital heart failure	18 977 (12.3)	11 005 (15.3)	13 813 (10.5)	8292 (12.9)	35 094 (8.7)	18 826 (10.4)
In-hospital mortality	3396 (1.9)	2221 (2.7)	2738 (1.9)	1782 (2.5)	7656 (1.8)	4802 (2.5)

Abbreviations: ACS, acute coronary syndrome; DAPT, dual antiplatelet therapy; NSTE, non–ST-segment elevation; onset-EMS, time from symptom onset to calling emergency medical service; onset-FMC, time from symptom onset to first medical contact; PCI, percutaneous coronary intervention; STEMI, ST-segment elevation myocardial infarction.

^a^ Unless otherwise indicated, data are expressed as No. (%) of patients. Denominators were varied according to the different missing rates of outcome variables (eTable 3 in Supplement 1). Data for DAPT and statin discharge were collected from June 2019 to December 2020.

**Figure 1.**
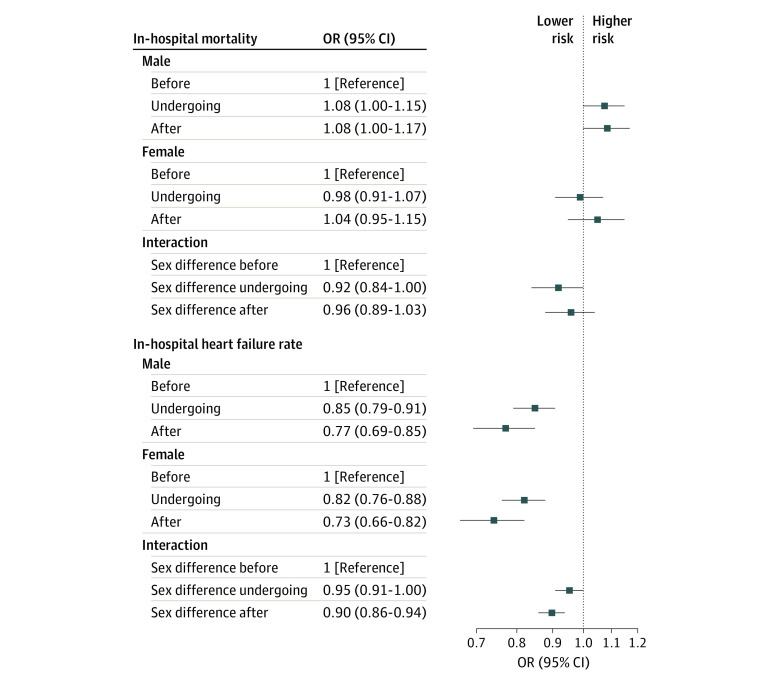
Association of the National Chest Pain Center Program With Sex Differences in Treatment Outcomes for Patients With Acute Coronary Syndrome Patient groups are classified as before the date of accreditation (≥6 months), undergoing accreditation (during the 6 months before accreditation), and after the date of accreditation. OR indicates odds ratio.

The onset-FMC time was lower with ACS for men in the undergoing accreditation (β coefficient, −0.07 [95% CI, −0.09 to −0.05]) and after accreditation (β coefficient, −0.06 [95% CI, −0.09 to −0.02]) groups and for women in the undergoing accreditation (β coefficient, −0.10 [95% CI, −0.13 to −0.06]) and after accreditation (β coefficient, −0.08 [95% CI, −0.12 to −0.03]) groups when compared with the before accreditation group. Interaction analysis showed that the sex-related difference in onset-FMC time for ACS was smaller in the undergoing accreditation (β coefficient, −0.04 [95% CI, −0.06 to −0.02]) and after accreditation (β coefficient, −0.03 [95% CI, −0.04 to −0.01]) groups than in the before accreditation group. Compared with the before accreditation group, there was no significant change in sex-related difference in onset-EMS and non-PCI length of stay in the undergoing accreditation and after accreditation groups (Figure 1).

**Figure 2.**
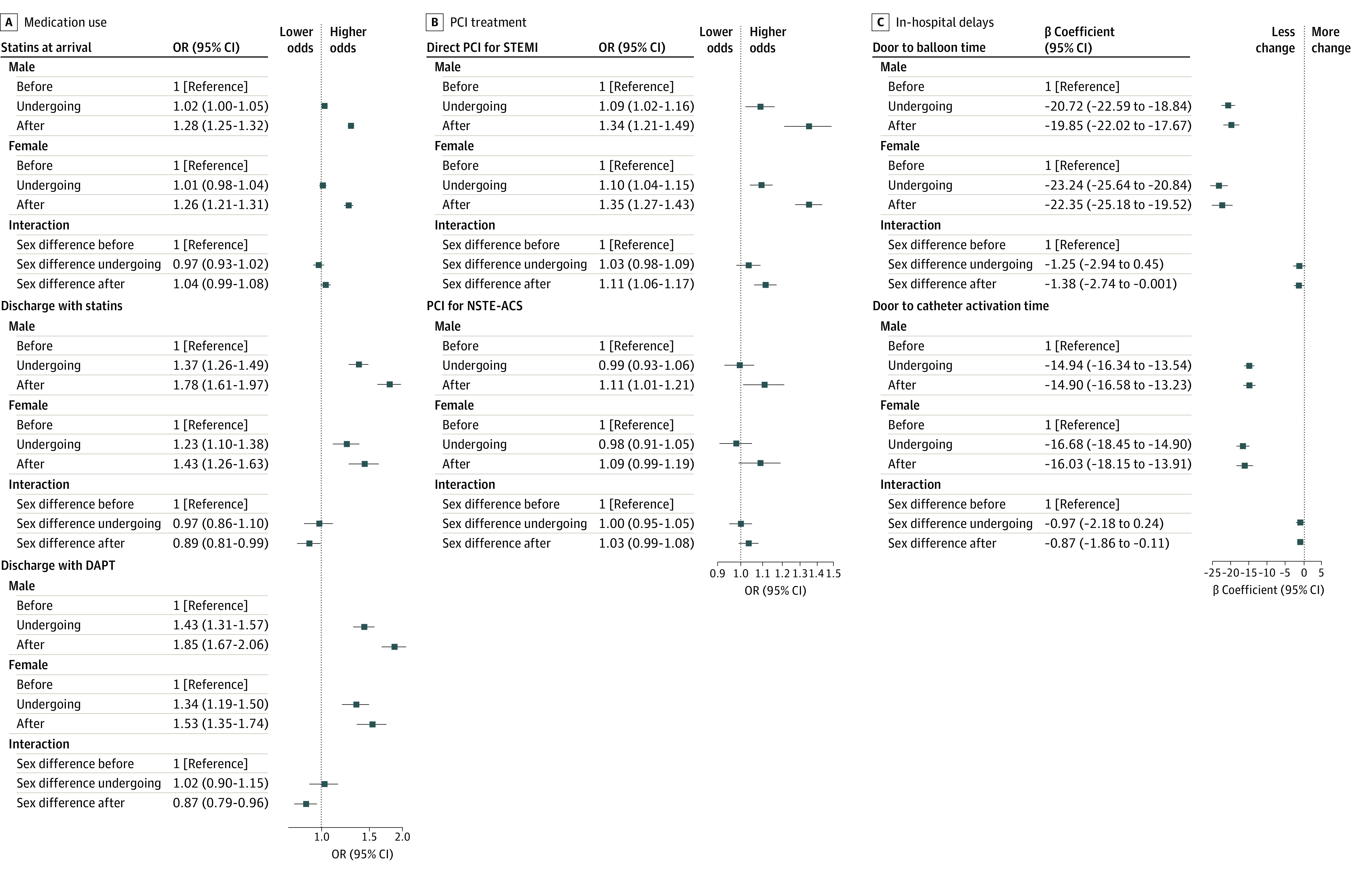
Association of the National Chest Pain Center Program With Sex Differences in In-Hospital Treatment for Patients With Acute Coronary Syndrome (ACS) Patient groups are classified as before the date of accreditation (≥6 months), undergoing accreditation (during the 6 months before accreditation), and after the date of accreditation. DAPT indicates dual antiplatelet therapy; NSTE, non–ST-segment elevation; OR, odds ratio; PCI, percutaneous coronary intervention; and STEMI, ST-segment elevation myocardial infarction.

The rate of statin use at arrival was higher in both men (odds ratio [OR] 1.28 [95% CI, 1.25-1.32]) and women (OR 1.26 [95% CI, 1.21-1.31]) in the after accreditation group than in the before accreditation group. There was no significant change in the sex-related difference in statin use after admission in the after accreditation groups. The difference in discharge with statin (OR, 0.89 [95% CI, 0.81-0.99]) and discharge with DAPT (OR, 0.87 [95% CI, 0.79-0.96]) between men and women was higher in the after accreditation group than in the before accreditation group. The difference between the proportions of men and women with STEMI who underwent direct PCI was smaller in the after accreditation group than in the before accreditation group (OR, 1.11 [95% CI, 1.06-1.17]). The sex-related difference in door to balloon time in the after accreditation group was smaller in patients with ACS (β coefficient, −1.38 [95% CI, −2.74 to −0.001]) than in the before accreditation group (Figure 2).

**Figure 3.**
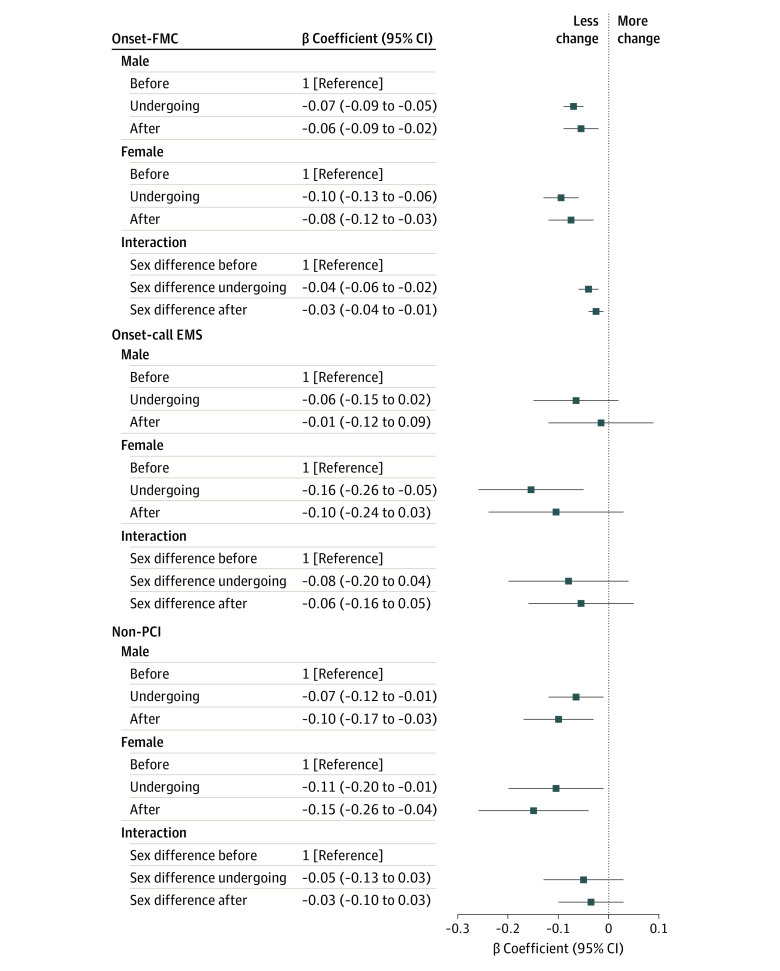
Association of the National Chest Pain Center Program With Sex Differences in Prehospital Delays for Patients With Acute Coronary Syndrome Patient groups are classified as before the date of accreditation (≥6 months), undergoing accreditation (during the 6 months before accreditation), and after the date of accreditation. Non-PCI indicates hospital stay without percutaneous coronary intervention; onset-EMS, time from symptom onset to calling emergency medical service; and onset-FMC, time from symptom onset to first medical contact.

The proportion of men and women with in-hospital heart failure was lower in the undergoing accreditation and after accreditation groups than in the before accreditation group. The sex-related difference was smaller in the undergoing accreditation (OR, 0.95 [95% CI, 0.91-1.00]) and after accreditation (OR, 0.90 [95% CI, 0.86-0.94]) groups than in the before accreditation group. There was no significant change in the sex-related difference in in-hospital mortality in the undergoing accreditation (OR, 0.92 [95% CI, 0.84-1.00]) and after accreditation (OR, 0.96 [95% CI, 0.89-1.03]) groups compared with before accreditation group (Figure 3).

A sensitivity analysis using IPTW was performed to confirm the accuracy of the estimated coefficients, and the results were consistent with those of the primary model. The NCPCP was associated with lower differences in the onset-FMC time, direct PCI rate, in-hospital rate of heart failure, and time from door to balloon for male and female patients with ACS (eTable 4 in Supplement 1).

## Discussion

As reported previously,^37,38,39,40,41^ this cross-sectional study found significant sex-specific differences in health care delivery and outcomes in patients with ACS. Compared with previous studies, we found the absolute sex differences in reperfusion therapy and medical therapies in the before accreditation group did not change over time, which further underscored the need to specifically address these differences with quality improvement programs.^13,37^ These differences may reflect biological differences between men and women, the presence of elements such as prejudice, and outcomes of the interaction between the two.^42^

In previous evaluations,^25,28,43^ quality improvement programs were associated with reduced sex-related differences in at least 1 outcome, highlighting their potential to reduce differences in health care services offered to men and women. In this study, evaluation of the effect of the NCPCP on sex-specific disparities in patients with ACS reveals that the differences in onset-FMC time, direct PCI for STEMI, door to balloon time, and rate of in-hospital heart failure between men and women were smaller after NCPCP. We further confirm that sex disparities in health care could be reduced if clinicians were more compliant with clinical guidelines.

Potential reasons for the undertreatment of women with ACS have been explored previously. The atypical symptoms of ACS in women mean that fewer women than men undergo tests such as electrocardiography or arteriography, which delays diagnosis and acute treatment.^41,44^ In our study, compared with women, the men with ACS tended to have more persistent pain and/or tightness. Because of estrogen levels, women with ACS tend to be older and, therefore, have more comorbid conditions such as hypertension, type 2 diabetes, and heart failure, which result in more contraindications to ACS treatment.^45^ Elderly women with ACS often have type 2 diabetes, and the risk of bleeding after an invasive procedure is substantially higher in women than in men, leading to a lower PCI rate.^46,47^ Furthermore, women tend to be disadvantaged in terms of socioeconomic status and social support, which may contribute to health services being less accessible for women. Moreover, women in China are less proactive than men in terms of their health care behavior, resulting in delays in presentation to hospital.^39,48^ Additionally, we found that most cardiologists do not have a clear understanding of sex differences in treatment between men and women and lack additional attention to female ACS patients.

Our findings indicate that NCPCP was associated with lower sex-specific disparities in patients with ACS to some extent, and there are several potential reasons for these changes. First, the NCPCP highlights regional coordination of treatment and closer connection between in-hospital and prehospital emergency systems, reducing prehospital delays for patients with ACS and improving the treatment capacity of hospitals.^49^ Second, women with ACS tend to be older, are likely to present with comorbid diabetes and hypertension, and have atypical symptoms at onset. Creating CPC and following standardized treatment procedures can identify women with ACS more promptly and increase the use of guideline-recommended therapies. Third, health education at the community and patient levels has improved women’s awareness of ACS, such that women are more likely to seek medical treatment promptly when they have symptoms suggestive of ACS, which may reduce the sex-related differences in onset-FMC time.

Some of the indicators of sex-specific differences in quality of service for patients with ACS were lower after NCPCP, but the differences in in-hospital mortality and medication use between men and women remain unchanged. Multilevel factors are associated with disparities in the quality of health care services, including those at the system, organization, and individual levels.^50^ Interventions at multiple levels may be needed to reduce these disparities.^51^ The NCPCP’s focus on the community, organization, and individual levels further confirms the positive effects of regional interventions. Quality improvement programs need to incorporate the concept of sex-specific differences into interventions at multiple levels from a systemic perspective to increase awareness of clinicians.^52^ Quality improvement interventions should focus on vulnerable groups, including women. From interventions to performance reviews, more attention should be paid to improving disparities of service quality between men and women.

### Limitations

This study had some limitations. First, the data analyzed were obtained from the CCA Database–CPC. Given that they are reported data, the possibility of selection bias cannot be excluded. Second, we used a before-and-after control design, and parallel controls could not be set due to data limitations, which means that we could not fully examine any causal effect of the NCPCP. Third, the information about medical history and risk factors for cardiovascular diseases was missing in our study, which may bias the results. Finally, the study did not include long-term patient outcomes, so the effect of quality improvement on long-term sex-specific differences in the prognosis could not be evaluated.

## Conclusions

The findings of this cross-sectional study suggest that although the NCPCP was associated with lower disparities between men and women with ACS, these differences persist nonetheless. The NCPCP needs to further emphasize sex disparities to cardiologists; highlight compliance with clinical guidelines, especially for female patients; and include the reduction of sex disparities as one of the performance appraisal indicators.

## Notes

Supplement 1. **eFigure 1.** Distribution of Accredited Chest Pain Centers in China **eFigure 2.** Flowchart Showing the Data Selection Process **eTable 1.** CPC Accreditation and QI Initiatives in the NCPCP Program **eTable 2.** Definitions of Quality Indicators **eTable 3.** The Missing Rate of Outcome Variables **eTable 4.** Sensitivity Analysis of the Effect of the National Chest Pain Center Program on Sex-Specific Differences in Service Quality for Patients With ACS

Supplement 2.
Data Sharing Statement
