# Supplementary material for: Sex Disparities in Management and Outcomes Among Patients With Acute Coronary Syndrome
Source: JAMA Netw Open. 2023 Oct 20;6(10):e2338707. doi: 10.1001/jamanetworkopen.2023.38707 (PMC10589815; doi:10.1001/jamanetworkopen.2023.38707)
Supplement: Supplement 1. — eFigure 1. Distribution of Accredited Chest Pain Centers in China eFigure 2. Flowchart Showing the Data Selection Process eTable 1. CPC Accreditation and QI Initiatives in the NCPCP Program eTable 2. Definitions of Quality Indicators eTable 3. The Missing Rate of Outcome Variables eTable 4. Sensitivity Analysis of the Effect of the National Chest Pain Center Program on Sex-Specific Differences in Service Quality for Patients With ACS [file jamanetwopen-e2338707-s001.pdf]

Supplementary Online Content

Zhou S, Zhang Y, Dong X, et al. Sex disparities in management and outcomes among patients with acute coronary syndrome in China. *JAMA Netw Open*. 2023;6(10):e2338707. doi:10.1001/jamanetworkopen.2023.38707

eFigure 1. Distribution of Accredited Chest Pain Centers in China
eFigure 2. Flowchart Showing the Data Selection Process
eTable 1. CPC Accreditation and QI Initiatives in the NCPCP Program
eTable 2. Definitions of Quality Indicators
eTable 3. The Missing Rate of Outcome Variables
eTable 4. Sensitivity Analysis of the Effect of the National Chest Pain Center Program on Sex-Specific Differences in Service Quality for Patients With ACS

This supplementary material has been provided by the authors to give readers additional information about their work.

© 2023 Zhou S et al. *JAMA Network Open*.

eFigure 1. Distribution of Accredited Chest Pain Centers in China

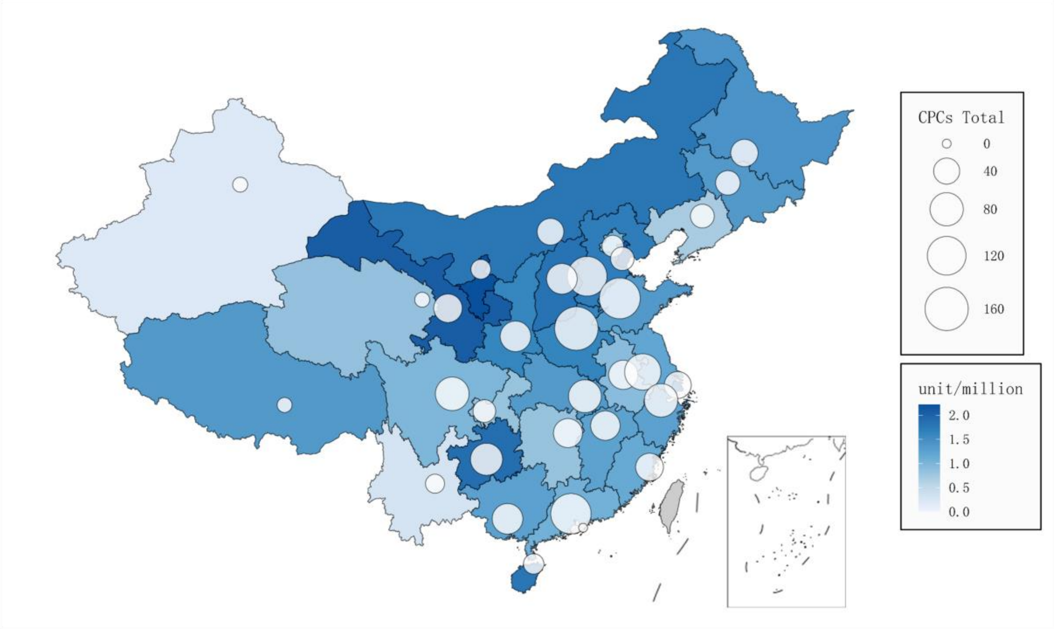

© 2023 Zhou S et al. *JAMA Network Open*.

eFigure 2. Flowchart Showing the Data Selection Process

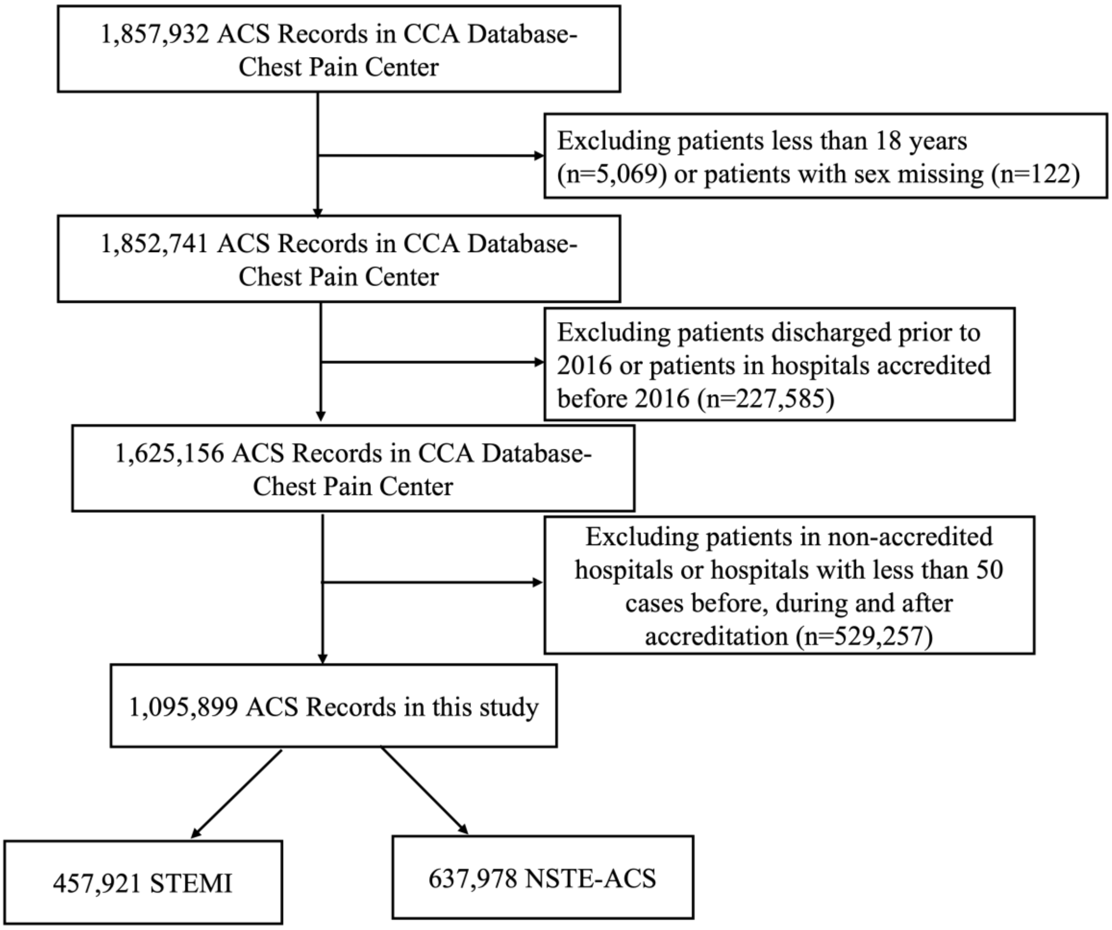

eTable 1. CPC Accreditation and QI Initiatives in the NCPCP Program

Interventions	Details
1) Accreditation of hospital-based CPCs	▪ The comprehensive center criteria are applicable to the comprehensive CPCs at tertiary hospitals; while the basic center criteria targets at the basic CPCs at secondary hospitals. The basic CPCs can choose any of the three reperfusion

© 2023 Zhou S et al. *JAMA Network Open*.

	therapies, including emergency PCI, transfer-in PCI or thrombolysis, as the priority for ACS patients.<br>▪ Both editions of the criteria include 5 dimensions of qualification: conditions of facilities, diagnosis and treatment process, integration of prehospital and hospital care, training and education, and real-time data reporting.<br>▪ Registered hospitals need to go through 3 stages including self-assessment, accreditation and re-accreditation every 3 years, to develop an accredited CPC. The registered CPC should report data in real time to China CPC Data Reporting Platform for more than 6 months before starting accreditation.<br>▪ The accreditation is based on a review of information from multiple sources, including self-assessment statements, real-time data reporting and field survey. The accreditation process is jointly led by the China CPC Headquarters, Regional Accreditation Offices, and Provincial-level CPC Alliances. The Accreditation Working Committee has the final decision on whether or not to approve the accreditation application.
2) Performance measure and assessment	▪ Accredited hospitals should continuously report data for monitoring and feedback.<br>▪ The indicators for measuring the CPC performance in the quarterly and annually benchmarked reports are developed by the China CPC Headquarters, based on the ACC/AHA Performance Measures and clinical practice guidelines. There are two sets of performance measures respectively for comprehensive and basic CPCs.<br>▪ The QI Center create practical tools for sites to help improve the quality of data reporting among the participant hospitals. The tools include guidelines for data reporting, a set of internal quality assurance tools, and a yearly data audit program.<br>▪ Ranking of a CPC is calculated based on the percentile of each indicator and a weighted composite score. The score of 100, 80, 60, 40, 20 and 0 are for ranking the top 10%, 10-30%, 30-50%, 50-70%, 70-90% and 90-100% of the measure among the entire accredited CPCs.
3) Quality audit and feedback	▪ Improvement in adherence to the guideline recommendations is facilitated through monthly and quarterly hospital-specific performance feedback reports.<br>▪ The hospital-specific data are compared against a variety of internal and external benchmarks, including the temporal trend in performance and comparison points to regional or national performance thresholds.<br>▪ A series of regular meeting, QI analysis meeting and case study meeting are carried out at least once every quarter for sharing of 'best practice' clinical support tools.<br>▪ The QI Center create practical tools for sites to help improve the quality of data reporting across the participant hospitals. The tools include guidelines for data reporting, monthly checklists for invalid and illogical data errors, and a yearly data audit program.
4) Education and training	▪ The CCA conducts routine training programs, which are mainly aimed at healthcare professionals at CPCs.

© 2023 Zhou S et al. *JAMA Network Open*.

	▪ The contents of training include clinical skills for the diagnosis and treatment of ACS, partnership as a multidisciplinary team, and reporting of clinical data for performance measure and quality audits.<br>▪ Education for residents in the community where the hospital is located.<br>▪ Frequencies of website visits and downloads are tracked to evaluate the outreach and engagement activities of each registered hospital.

Abbreviations: QI: quality improvement; NCPCP: National Chest Pain Centers Program; PCI: percutaneous coronary intervention; CPC: chest pain center; EMS: emergency medical services; ACC/AHA: American College of Cardiology/American Heart Association; ACS: acute coronary syndromes.

© 2023 Zhou S et al. *JAMA Network Open*.

eTable 2. Definitions of Quality Indicators

	Indicators	Definitions
Pre-hospital indicators	Onset to first medical contact	Time from ACS patients onset to first medical contact
	Onset to calling EMS	Time from ACS patients onset to call emergency health cares
	Non-PCI hospital stay time	Time delay of ACS patients in non-PCI capable hospitals
In-hospital management process indicators	Statins at arrival	ACS patients with indications receiving statins within 24 hours after hospital arrival
	Direct PCI for STEMI	Proportion of STEMI patients undergoing direct PCI
	PCI for NSTE-ACS	Proportion of patients with moderate-to-high-risk NSTE-ACS receiving PCI
	Time from door to catheterization activation	Time from hospital door to catheterization lab activation for ACS patients
	Time from door to balloon	Time from hospital door to balloon for ACS patients
	Discharge with Statin	Proportion of ACS patients who are prescribed statins at discharge
	Discharge with DAPT	Proportion of ACS patients who are prescribed dual antiplatelet therapy at discharge
Treatment outcome indicators	In-hospital mortality	Proportion of patients who died in hospitals
	In-hospital heart failure rate	Proportion of patients with new heart failure during hospitalization

ACS, acute coronary syndrome; DAPT, dual antiplatelet therapy; EMS, emergency medical service; PCI, percutaneous coronary intervention; STEMI, ST-segment elevation myocardial infarction; NSTE-ACS, non-ST-segment elevation acute coronary syndrome

© 2023 Zhou S et al. *JAMA Network Open*.

eTable 3. The Missing Rate of Outcome Variables

Outcome variables	Missing numbers, N	Missing rate,%
Onset to first medical contact	35,719	3.26
Onset to calling EMS	25,543	26.56
Non-PCI hospital stay time	24,637	13.45
Statins at arrival	177,286	16.18
Direct PCI for STEMI	42,713	9.33
PCI for higher risk of NSTE-ACS	53,680	8.41%
Time from door to catheterization activation	16,001	4.76
Time from door to balloon	15,379	4.92
Discharge with Statin	74,368	17.90
Discharge with DAPT	74,155	17.85
In-hospital mortality	1,702	0.16
In-hospital heart failure rate	90,535	8.26

eTable 4. Sensitivity Analysis of the Effect of the National Chest Pain Center Program on Sex-Specific Differences in Service Quality for Patients With ACS

		Undergoing accreditation		After accreditation	
	Pre-accreditation difference	OR/ Coefficient	95%CI	OR/ Coefficient	95%CI
PCI for STEMI	Ref	1.04	[0.98, 1.10]	1.11***	[1.06, 1.17]
Onset to FMC time	Ref	0.84***	[0.80, 0.89]	0.91**	[0.87, 0.96]
Time from door to balloon	Ref	-1.38	[-3.07, 0.31]	-1.48**	[-2.85, -0.12]
In-hospital heart failure rate	Ref	0.95**	[0.91,1.00]	0.90**)	[0.86, 0.95]

CI, confidence interval; OR, odds ratio; PCI, percutaneous coronary intervention; STEMI, ST-segment elevation myocardial infarction. **p<0·05, ***p<0·01

© 2023 Zhou S et al. *JAMA Network Open*.
