# Supplementary material for: Sex Disparities in Management and Outcomes Among Patients With Acute Coronary Syndrome
Source: JAMA Netw Open. 2023 Oct 20;6(10):e2338707. doi: 10.1001/jamanetworkopen.2023.38707 (PMC10589815; doi:10.1001/jamanetworkopen.2023.38707)
Supplement: Supplement 2. — Data Sharing Statement [file jamanetwopen-e2338707-s002.pdf]

# Data Sharing Statement

Zhou. Sex Disparities in Management and Outcomes Among Patients With Acute Coronary Syndrome in China. *JAMA Netw Open*. Published October 20, 2023.
doi:10.1001/jamanetworkopen.2023.38707

**Data**
**Data available:** No

**Additional Information**
**Explanation for why data not available:** Please contact the corresponding author for more information.
